# Overview of Antimicrobial Resistant ESKAPEE Pathogens in Food Sources and Their Implications from a One Health Perspective

**DOI:** 10.3390/microorganisms12102084

**Published:** 2024-10-18

**Authors:** Naomi Oyenuga, José Francisco Cobo-Díaz, Avelino Alvarez-Ordóñez, Elena-Alexandra Alexa

**Affiliations:** 1 School of Food Science and Environmental Health, Technological University Dublin, D07 H6K8 Dublin, Ireland; c18310063@mytudublin.ie; 2 Department of Food Hygiene and Technology, Universidad de León, 24071 León, Spain; jcobd@unileon.es (J.F.C.-D.); aalvo@unileon.es (A.A.-O.); 3 Institute of Food Science and Technology, Universidad de León, 24007 León, Spain

**Keywords:** antimicrobial resistance, multidrug, pathogenic organisms, food, food related reservoirs, surveillance, societal burden, human health

## Abstract

Antimicrobial resistance is an increasing societal burden worldwide, with ESKAPEE (*Enterococcus faecium*, *Staphylococcus aureus*, *Klebsiella pneumoniae*, *Acinetobacter baumannii*, *Pseudomonas aeruginosa*, *Enterobacter species* and *Escherichia coli*) pathogens overwhelming the healthcare sectors and more recently becoming predominantly a concern for their persistence in food and food industries, including agricultural settings and animal husbandry environments. The aim of this review is to explore the mechanisms by which the ESKAPEE group gained its multidrug resistance profiles, to analyse their occurrence in different foods and other related reservoirs, including water, and to address the current challenges due to their spread within the food production chain. Moreover, the repertoire of surveillance programmes available focused on monitoring their occurrence, common reservoirs and the spread of antimicrobial resistance are described in this review paper. Evidence from the literature suggests that restricting our scope in relation to multidrug resistance in ESKAPEE pathogens to healthcare and healthcare-associated facilities might actually impede unveiling the actual issues these pathogens can exhibit, for example, in food and food-related reservoirs. Furthermore, this review addresses the need for increasing public campaigns aimed at addressing this challenge, which must be considered in our fight against antimicrobial resistance shown by the ESKAPEE group in food and food-related sectors.

## 1. Introduction

Antimicrobial resistance (AMR) has emerged as a critical global public health concern, as recognised by the World Health Organization (WHO), the European Centre for Disease Prevention and Control (ECDC), and the United States Centers for Disease Control and Prevention (CDC), for which finding new medicines to fight against is paramount to avoid the prediction of 40 million deaths annually by 2050 [1,2,3]. Indeed, in 2019, antibiotic resistance was thought to have contributed to 4.95 million fatalities worldwide, of which 1.27 of them were directly linked to bacterial AMR [4]. This has become an enduring issue as there are limited, or in some cases, no effective antimicrobial treatments available for diseases caused by antimicrobial-resistant bacteria [5]. The evolution of bacterial pathogens acquiring resistance to various antimicrobials, classified as multidrug-resistant (MDR) bacteria, is challenging antibiotic therapy. In 2017, the WHO published a list of the most critical antibiotic-resistant pathogens for which urgent attention is required and placed these pathogens, among others, as most critical from a clinical perspective. The ESKAPEE group includes seven clinically significant MDR microorganisms: *Enterococcus faecium*, *Staphylococcus aureus*, *Klebsiella pneumoniae*, *Acinetobacter baumannii*, *Pseudomonas aeruginosa*, *Enterobacter* species and *Escherichia coli* [2]. The acronym “ESKAPEE” refers to these bacteria as they can ‘escape’ the effects of standard antibiotics, thus making them fatal adversaries, especially in clinical settings [6]. Commonly available treatment options, including broad-spectrum beta-lactams, carbapenems, glycopeptides, fluoroquinolones and aminoglycosides, are currently being challenged by the ESKAPEE pathogens [2,7].

According to the latest findings by Murray et al. [4], AMR burden, in terms of deaths, can be mostly attributed to methicillin-resistant *S. aureus*, accounting for 100,000 deaths, followed by fluoroquinolone-resistant *E. coli*, carbapenem-resistant *A. baumannii* or *K. pneumoniae*, or *K. pneumoniae* resistant to third-generation cephalosporins, causing from 50,000 to 100,000 deaths per year each [4]. The increased number of bloodstream infections in hospital environments is worrying, based on the latest AMR surveillance report [8], driven by *K. pneumoniae* resistant to third-generation cephalosporins and *K. pneumoniae* and *Acinetobacter* spp. resistant to carbapenems [8]. Regarding the food sector, ESKAPEE pathogens may inhabit different compartments of the food production chain and pose a significant threat to public health, driven by the use of antibiotics in livestock, which promotes the development of resistant bacteria in food-producing animals. This issue is particularly critical given that resistant bacteria can transfer to humans through the food supply, leading to more difficult-to-treat bacterial-associated infections. Overuse or misuse of antibiotics in animal husbandry could increase resistance in bacteria, thus promoting their spread throughout food production and consumption [9,10]. The widespread use of antibiotics for prophylactic purposes in animal husbandry accelerated the emergence of resistant bacteria, of which *E. coli* and *E. faecium* showed resistance gene patterns associated in humans with difficulties in treating infections with standard antibiotics [11]. This review addresses the evolution and emergence of ESKAPEE pathogens in food and drinking water and summarises the current antibiotic treatments available for them and the mechanisms of acquired AMR in these pathogens. Future strategies to combat resistance in ESKAPEE pathogens, as well as different surveillance programmes available, are also discussed in the present review study.

## 2. Current Antibiotics for ESKAPEE Pathogens, Alternatives and Their Mechanisms of Action

Different antibiotics have specific target sites to counteract the action of bacterial pathogens, including inhibiting bacterial cell wall synthesis, inhibition of protein synthesis, altering nucleic acids, disrupting membranes or functioning as anti-metabolites [12].

**Glycopeptides and alternatives**. Different pathogen-drug combinations are employed to combat the action of ESKAPEE pathogens, of which vancomycin is listed as an antibiotic to treat infections caused by methicillin-resistant *S. aureus* (MRSA) and/or *E. faecium*. Its mode of action is to prevent cell wall synthesis in Gram-positive bacteria by binding to the D-alanyl-D-alanine precursors [13,14]. As a consequence, the peptidoglycan chains cannot cross-link, thus weakening the bacterial cell walls, which eventually allows intracellular components to leak out, leading to the death of the bacterial cell [13,14]. Alternatives to glycopeptides include linezolid and daptomycin, which are critical antibiotics used in the treatment of infections caused by vancomycin-resistant *E. faecium* (VRE), a pathogen known for its resistance to multiple antibiotics and its role in severe nosocomial infections [15,16]. Similarly, the increased effectiveness of daptomycin makes it a good alternative for the treatment of MRSA bloodstream infections [15]. Linezolid, from the oxazolidinones family, binds to a specific site of the 23S rRNA 50S subunit, preventing the functional 70S initiation complex required for bacterial protein synthesis [12]. On the other hand, daptomycin belongs to the lipopeptides class of antibiotics and has a distinct mechanism of action that involves disrupting the bacterial cell membrane, thus inhibiting the peptidoglycan synthesis and/or lipoteichoic acid synthesis [17].

**Carbapenems and alternatives**. These antibiotics belong to the beta-lactams family and inhibit bacterial cell wall synthesis. Some carbapenems, such as imipenem and meropenem, are utilised to treat infections caused by *P. aeruginosa* and Enterobacterales species, including *K. pneumoniae* [18]. Carbapenems exert their antibacterial effects by irreversibly binding to the active site of the penicillin-binding proteins (PBPs), preventing the formation of a functional cell wall, thus leading to cell lysis and death [18]. Polymyxins are listed as critical treatment options for infections caused by MDR Gram-negative bacteria, of which colistin is considered a last resort antimicrobial to treat infections caused by carbapenem-resistant *A. baumannii* and *P. aeruginosa* [19,20]. They interact with the lipopolysaccharides in the outer membrane of Gram-negative bacteria, displacing calcium and magnesium ions that stabilise the membrane [19]. This interaction disrupts the integrity of the cell membrane, leading to increased permeability, leakage of cellular contents, and, ultimately, bacterial cell death [19]. Moreover, tigecyclines have exhibited robust activity against carbapenem-resistant *K. pneumoniae*, making it a promising alternative to treat various infections, although emerging resistance to these antibiotics has been noticed [21,22].

**Cephalosporins**. These are beta-lactam antibiotics that inhibit bacterial cell wall synthesis with a mode of action similar to that of carbapenems. The bacterial cell wall becomes vulnerable to osmotic pressure and autolysis, leading to a weakened cell wall and eventually death [12]. Cephalosporins are categorised into different generations based on their spectrum of activity, with each generation offering varying degrees of effectiveness against pathogenic bacteria [23].

**Fluoroquinolones**. These are synthetic antibiotics that inhibit bacterial DNA replication. This occurs by binding to bacterial topoisomerase II (DNA gyrase) and topoisomerase IV, enzymes essential for the unwinding and supercoiling of DNA during replication [24]. Inhibition of these enzymes prevents DNA replication, transcription, and repair, leading to bacterial cell death [24].

## 3. Mechanisms of Antimicrobial Resistance in ESKAPEE Pathogens

Through genetic mutations and/or acquired resistance genes on mobile genetic elements (MGEs), ESKAPEE pathogens have established mechanisms of resistance against oxazolidinones, lipopeptides, macrolides, fluoroquinolones, tetracyclines, beta-lactams, beta-lactam–beta-lactamase inhibitor combinations, and antibiotics that are the last line of defence, such as carbapenems, glycopeptides, and polymyxins [6]. Different mechanisms through which ESKAPEE pathogens can get away from the action of antibiotics include (1) modification of drug binding sites, (2) drug inactivation, (3) drug uptake reduction and (4) drug efflux pumps.

**Modification of drug binding sites.** To avoid being identified by antimicrobial drugs, several resistant bacteria modify their target sites. For example, a mutation in the gene encoding for unique PBPs, which are the enzymes involved in the formation and control of the cell wall peptidoglycan synthesis, results in a modified PBP2′ or PBP2a encoded by the *mec*A or *mec*C genes in the case of MRSA strains, thus rendering low affinity for beta-lactam drugs [25,26]. Other examples refer to changes in DNA gyrase or topoisomerase IV, encoded by *gyrA* and *parC* genes, respectively, thus leading to resistance mutations corroborated with resistance to fluoroquinolones [24].

**Drug inactivation**. Different enzymes can inactivate or degrade antibiotics, including beta-lactamases, aminoglycoside-modifying enzymes or chloramphenicol acetyltransferases. Through hydrolysation, tetracycline action can be avoided by the expression of *tetX* genes [27].

**Drug uptake reduction**. The susceptibility of bacteria to a particular drug is controlled by the balance between antibiotic absorption and excretion. Consequently, reducing the number of antibiotic molecules that may pass through their cell membrane is one-way bacteria could exploit to become resistant to the action of antibiotics [12,28]. The outer membrane of Gram-negative ESKAPEE bacteria contains membrane transporter proteins called porins, allowing antibiotics to pass through. However, a porin loss or mutation may occur, allowing bacteria to develop resistance to particular antibiotics, as it occurs with the reduced susceptibility of *K. pneumoniae* strains to beta-lactams [28].

**Efflux pumps**. Membrane transporters can act as efflux pumps, which can expel drugs from the cell quickly. Therefore, the concentrations of antibiotics used must be adequate to achieve an antibacterial outcome [28]. Six superfamilies of efflux pumps have been described based on their structure and their energy requirements [20,29], which include the (1) adenosine triphosphate (ATP)-binding cassette (ABC) family, directly linked to using ATP as an energy source, while the other five act as secondary transporters: (2) multidrug and toxin extrusion (MATE) group, (3) major facilitator superfamily (MFS), (4) resistance-nodulation-cell division (RND) superfamily, (5) small multidrug resistance (SMR) family and the (6) proteobacterial antimicrobial compound efflux (PACE) family [29]. For example, MexAB-OprM and MexCD-OprJ are involved in the survival of *P. aeruginosa* in the presence of toxic substances, having the capacity to develop resistance to at least three main classes of antibiotics: aminoglycosides, carbapenems, and fluoroquinolones [28,30].

Another mechanism of resistance involves the **development of biofilms** in ESKAPEE pathogens, which directly lowers the efficiency of antibiotics. These circumstances may hinder the effectiveness of antimicrobial agents, resulting in increased bacterial tolerance to the nutritional shortage, making the cells in deeper layers of biofilms become more resistant to the action of antibiotics [31]. Adhesion signaling in ESKAPEE pathogens to biotic and abiotic surfaces initiates the biofilm formation that needs to adapt to different environmental stresses with the acquisition of genetic information resulted from different horizontal gene transfer events, therefore directly influencing the antimicrobial resistance profiles of ESKAPEE pathogens [31].

## 4. ESKAPEE Pathogens in Food and Water Sources

Antibiotics are extensively used in animal husbandry and veterinary medicine to prevent or treat disease, leading to the selection of resistant bacteria in livestock. These resistant bacteria can enter the food chain through the consumption of contaminated meat, milk, eggs, and products thereof [32]. For instance, *E. coli* and *K. pneumoniae*, commonly found in livestock, can acquire resistance genes and transfer them to humans *via* food [32]. Globally, the livestock farming sector uses 73% of all available antibiotics [33], but it is believed that other associated environments are contributing to increased resistance in bacterial pathogens. For instance, the use of slurry and manure in agriculture may also lead to increased spread of resistance genes to animals, humans, and aquatic habitats [33]. Manure spread onto the fields increased the reservoir of clinically important antibiotic resistance genes and resistant bacteria when compared with other fertilisers, leading to the absorption of these into food crops [33]. Other factors involved would include climatic conditions and cross-contamination events, especially if improper manufacturing and hygiene practices are followed [34]. A plethora of studies have investigated the presence and characterised multidrug resistance profiles of ESKAPEE pathogens in food and water sources, which are presented below.

### 4.1. Prevalence of ESKAPEE Pathogens in Food Sources

A study performed by Pesavento et al. [35] found that 35.5% of raw meat samples (beef, poultry and pork) and 44.9% of ready-to-eat (RTE) products (cheese, salads and ham) from Italian retail markets were contaminated with *E. faecium*. Among these, 22.1% of isolates harboured resistance to erythromycin, followed by resistance to tetracyclines (16.4%), gentamicin (13.6%) and in a lesser extent to ciprofloxacin (10.7%) [35]. Similarly, a study conducted in Turkey by Sanlibaba et al. [36] reported a 61.9% prevalence of *E. faecium* in pre-packaged chicken meat samples. Increased resistance among the *E. faecium* isolates was registered, of which resistance to rifampicin prevailed, accounting for 81.7% of isolates, followed by resistance to ampicillin (73.3%), erythromycin (45%) and 31.7% to ciprofloxacin [36]. More recently, 31.6% of fermented milk products from Poland were contaminated with *E. faecium* [37]. Regarding resistance profiles, 29.7% of isolates showed resistance to streptomycin, followed by resistance to erythromycin (14.9%) and tetracyclines (10.9%). These results raised concern as these food products were frequently contaminated with MDR *E. faecium* strains [37].

Regarding *S. aureus*, dairy product contamination is frequently reported, being present in cheese (27.8%), traditional ice cream (9.1%), and traditional butter (8.1%) [38]. MDR accounted for 45% of the *S. aureus* isolates, showing resistance to ampicillin (55%), tetracyclines (40%) and penicillin G (30%) [38]. Additionally, Wu et al. [39] revealed that 35% of 1850 retail meat and meat products analysed were contaminated with *S. aureus*. The most common resistance found was to ampicillin (85.4%), then penicillin (84.6%), erythromycin (52.7%), tetracycline (49.3%), kanamycin (45.3%), telithromycin (30.1%), clindamycin (29.6%) and streptomycin (21.1%) [39]. Similarly, raw chicken meat was contaminated with *S. aureus* strains resistant to cefpodoxime and cloxacillin (100% of isolates), ceftazidime and piperacillin/tazobactam (92.5%), clindamycin (72.5%), cefoxitin and vancomycin (70%), ofloxacin (67.5%), gentamicin (60%), methicillin (57.5%), and azithromycin (47.5%) [40].

Another important ESKAPEE pathogen to mention is *K. pneumoniae*, which the most recent prioritisation by the World Health Organization placed at the top of the list, referring to carbapenem-resistant *K. pneumoniae* as the main concern [1]. Gundogan et al. [41] revealed that 46.7% of raw calf meat and chicken samples were contaminated with *K. pneumoniae*, of which 100% of isolates showed resistance to ampicillin and amoxicillin and only 29% of isolates to aztreonam [41]. The prevalence rates in other food commodities showed *K. pneumoniae* isolates had resistance to 16 out of 21 antimicrobials tested [42]. In this study, various food products, including fish, shrimp, raw chicken meat, frozen goods, and cooked foods such as meat, vegetables, flour, and rice products, were analysed, with fresh raw poultry registering the highest contamination by *K. pneumoniae* (13.8%), followed by frozen raw food (11.4%), fresh raw seafood (8.2%) and cooked foods (7.5%) [42]. Resistance to ampicillin (92.3%), tetracycline (31.3%), trimethoprim-sulfamethoxazole (18.2%), and chloramphenicol (10.1%) was observed, therefore accounting towards an increased trend in MRD *K. pneumoniae* strains, mainly associated with fresh raw chicken (50%) [42]. On the other hand, cooked food samples from university campuses were tested for *K. pneumoniae*, including cooked rice, cassava couscous (attiéké), fried fish, fish soup, and raw fresh vegetables [43]. A prevalence of 15% of *K. pneumoniae* strains, among 160 food samples tested, was found, with resistance to amoxicillin (92.3%) being the most prevalent phenotype registered, followed by resistance to other beta-lactams [43].

In a study conducted by Askari et al. [44], *A. baumannii* was found in approximately 20% of the 194 raw meat samples investigated. Among the antibiotics screened, increased resistance to gentamicin (87.2%), tetracycline (79.5%), erythromycin (74.4%), azithromycin (66.7%), ciprofloxacin (59%), trimethoprim/sulfamethoxazole (56.4%) and rifampicin (51.3%), was exhibited by these isolates. Although less prevalent, resistance to imipenem (17.9%) and chloramphenicol (28.2%) was also noticed [44]. Similarly, milk samples contaminated with *A. baumannii* were resistant to beta-lactams, notably cefotaxime (44%), ampicillin-sulbactam and levofloxacin (33.3%), imipenem, meropenem, and aztreonam (22.2%) [45].

Twenty-nine out of 370 samples comprising raw, frozen, and imported bovine meat, along with various meat products such as burgers, kebabs, sausages, and salami, were contaminated with *P. aeruginosa* [46]. This study revealed high resistance rates to several antibiotics, including ampicillin (89.6% of the isolates), penicillin (86.2%), tetracycline (82.7%), gentamicin (51.7%), and cefoxitin (37.9%) [46]. In agreement with these findings, similar resistance profiles were noticed for isolates from milk and dairy-associated products (Kareish cheese, Damietta cheese, and plain yoghurt) in another study conducted in Egypt [47]. In this instance, all strains showed resistance to amoxicillin-clavulanic acid, clindamycin, vancomycin and lincomycin, and other resistances were noticed, such as to erythromycin and oxacillin (95.5%) and colistin (91%). In another instance, a study performed in fruits and vegetables led to the isolation of *P. aeruginosa* strains showing resistance to ampicillin (100% of the isolates), chloramphenicol (84%) and sulfamethoxazole/trimethoprim (83%) [48]. Although the occurrence of *P. aeruginosa* was investigated by various researchers, less frequent in terms of acquiring MDR was shown to be associated with fruits and vegetables, for example, among 163 *Pseudomonas* spp. analysed (recovered from 145 analysed samples: green beans, zucchini, cucumbers and others), only 37 *P. aeruginosa* isolates were recovered with no MDR profiles in place. In this instance, the MDR isolate investigated exhibited resistance to imipenem, doripenem, meropenem, ceftazidime, and aztreonam showed in *P. fluorescens* instead [49].

Lastly, other Enterobacterales and their associated antibiotic resistances, more specifically *Enterobacter* spp. and *E. coli*, have also received attention. Recently, Elsherbeny et al. [50] conducted a study to characterise the susceptibility to antimicrobials of Enterobacterales strains isolated in forty randomly selected RTE food samples (including dairy products and meats) in Egypt. 139 Enterobacterales (different species including *E. aerogenes*, *E. cloacae*, *Cronobacter sakazakii*) isolates were retrieved. The majority of isolates showed increased susceptibility to the antibiotics tested. However, increased resistance patterns for these particular species included resistance to third-generation cephalosporins and carbapenems, in addition to the more frequent ampicillin resistance profile [50]. Up to 274 meat-related food products were investigated for the presence of *Enterobacter* spp., including species such as *E. cloacae*, *E. hormaechei*, and *E. kobei* by Edris et al. [51]. Isolates were resistant to beta-lactams as well as to third-generation cephalosporins and, in the case of *E. hormaechei* and *E. kobei,* to glycopeptides [51]. Additionally, three *E. cloacae* isolates, and all *E. kobei* and *E. hormaechei* isolates, were assigned a MDR classification as they were resistant to at least three different antibiotic classes. Furthermore, one *E. cloacae* isolate was identified as extensively drug-resistant (XDR), as it was resistant to at least five antibiotic classes [51].

Focusing on *E. coli*, Menck-Costa et al. [52] carried out an investigation to isolate and identify extended-spectrum beta-lactamases (ESBLs)-producing *E. coli* strains from samples of beef, pork, and poultry, along with an analysis of their antimicrobial resistance profiles. A total of 450 meat samples (such as chicken, beef, and pork) were analysed, revealing a prevalence of 37% for *E. coli* displaying resistance to third-generation cephalosporins, with chicken samples (109 out of 150 samples) having the highest contamination level [52]. In addition to beta-lactams, there were other notable patterns of decreased susceptibility, with 51% of strains showing resistance to tetracycline, 46% to ciprofloxacin, and 38% to fosfomycin, although all *E. coli* isolates showed susceptibility to imipenem. Furthermore, 45% of the strains showed resistance to three or more antimicrobial classes, apart from beta-lactams [52]. Another study reported a total of 188 of the 556 milk and dairy samples (33.8%) were positive for *E. coli*, with the highest contamination occurring in bulk tank milk samples (47.4%), followed by cow milk samples (34.7%) [53]. The 42 tested isolates were resistant to tetracycline, ampicillin, and amoxicillin, with an overall MRD profile of 88.1% of the isolates [53]. Furthermore, the study also reported the presence of *E. coli* O157 isolate (0.2% of the isolates). On foods of no animal origin, *E. coli* was detected in 32% of 300 samples of vegetables and herbs analysed, such as spearmint, leaf lettuce, coriander, Chinese cabbage and cucumbers [54], with leaf lettuce as the most contaminated (36.7%). All *E. coli* isolates (n = 96) were resistant to penicillin (100%), and some isolates showed decreased susceptibility to ampicillin, tetracycline, and amoxicillin, with resistance rates of 31.3%, 31.3%, and 31.3%, respectively [54]. Summarised information from previously published articles in terms of MDR determinants in ESKAPEE pathogens associated with food is presented in Table 1.

### 4.2. Prevalence of ESKAPEE Pathogens in Water Sources

Contaminated water can serve as a reservoir and transmission route for ESKAPEE bacterial pathogens, impacting public health through direct consumption or indirect exposure, such as in the case of recreational water uses and agricultural activities. Antibiotic-resistant and virulent microorganisms can also contaminate the environment through hospital wastewater [69]. A study conducted by Gotkowska-Płachta [70] outlined the drug resistance and virulence of enterococci in water from rivers sampled downstream and upstream from the wastewater release point. A total of 283 enterococci strains were examined, of which 38.8% and 42.9% were identified as *E. faecium* coming from treated wastewater and hospital wastewater, respectively. Regardless of the sampling location, *E. faecium* was the predominant enterococci species in wastewater samples, with four MDR strains collected from downstream points of sampling. Besides, 50% and 83%, respectively, of the isolated *E. faecium* strains in wastewater samples were resistant to streptomycin and trimethoprim [70]. In a study carried out by Jannati et al. [71], wastewater samples from four hospitals in Iran were collected to check for the presence of *E. faecium*. Among the 97 *E. faecium* isolates obtained, 60%, 54% and 5% were resistant to ciprofloxacin, rifampicin and glycopeptides, respectively.

Regarding *S. aureus*, drinking water collected from reservoirs, taps, wells, and storage tanks was investigated by Adesoji et al. [72]. Among 45 *S. aureus* isolates obtained from water samples, all were resistant to cefuroxime and cloxacillin, 97.8% to ceftazidime and cefuroxime, 91.1% to erythromycin and 80% to ceftriaxone, making all isolated *S. aureus* strains MDR. Furthermore, the antibiotic resistance in *S. aureus* in raw and treated hospital wastewater was investigated by Akya et al. [73]. Among collected isolates (n = 60), 59 of them (98%) were MDR and showed resistance to penicillin, which was followed by azithromycin (93% of raw and 96% of treated sewage isolates) and clindamycin (90% of raw and 83% of treated sewage isolates) [73].

To ascertain the patterns of antibiotic susceptibility of *P. aeruginosa* and *K. pneumoniae* isolated from 28 water wells, Aromolaran and Amodu [74] conducted a study on water used mainly for drinking, cooking, and bathing. A total of 11 isolates (*P. aeruginosa*- 6 and *K. pneumoniae*- 5) were resistant to cefuroxime (100%). Some isolates were also resistant to ceftazidime (81.8%), ampicillin (72.2%), ciprofloxacin and cefuroxime (63.6% each), gentamicin and ofloxacin (54.5% each) [74]. Furthermore, Alawi et al. [75] used a citizen science approach to collect water samples from 49 households to check for the presence of antimicrobial-resistant bacteria in such residencies. A total of 536 isolates, including 464 Gram-negative and 72 Gram-positive bacteria, were found in 28 household samples. *E. coli* (n = 40 isolates) was among the microorganisms identified in 10% (n = 5) of households, with 60% of the isolates showing resistance to at least one antibiotic. Regarding *Enterobacter* spp., 13 of the isolates were identified as *E. cloacae,* and one was *E. hormaechei* [75], whereas, among *Acinetobacter* spp., only 3 isolates were identified as *A. baumannii*. Particularly, sixteen *E. coli* isolates from a unique household were resistant to various combinations of antibiotic classes, such as phenols and tetracyclines [75].

## 5. Consequences to Public Health of MDR in ESKAPEE Pathogens

Since, in many cases, isolates from ESKAPEE pathogens are MDR, they are associated with high mortality rates. For instance, *K. pneumoniae* and *A. baumannii* are associated with high mortality rates in intensive care units (ICUs) and among immunocompromised patients [76,77]. Infections caused by these pathogens often result in severe outcomes, such as prolonged hospitalisation, higher treatment failure rates and increased need for intensive care [78]. The antibiotic resistance exhibited by ESKAPEE pathogens significantly reduces the effectiveness of current treatments in which widely used beta-lactams to treat infections caused by *E. faecium,* and *S. aureus* become a challenge [79]. Carbapenems are considered last-resort antibiotics for treating MDR bacterial infections. However, carbapenem-resistant *A. baumannii* has emerged [79], questioning the need for new alternatives to kick in.

Furthermore, cephalosporins were once highly effective against a broad range of bacterial infections but are now challenged, for which improving their antimicrobial activity is of utmost importance [80]. ESBLs produced by *K. pneumoniae* can hydrolyse a wide range of cephalosporins, making these antibiotics ineffective for treating infections [57,81]. Consequently, there is an increased risk of incurable infections due to inefficient associated treatments; according to predictions by the Organisation for Economic Cooperation and Development, the resistance to antibiotics used as the final treatment option will have doubled its levels from 2005 to 2035 [1,8,82]. *S. aureus*, particularly MRSA, exemplifies this issue by causing many standard antibiotics, such as beta-lactams and cephalosporins, to be ineffective [83,84]. According to a review by Ventola [85], the rapid evolution of antibiotic resistance in these pathogens has outpaced the development of new antibiotics, leaving limited treatment options for healthcare providers. The presence of ESKAPEE pathogens complicates numerous medical procedures, particularly in surgical settings and the management of chronic diseases [86]. These pathogens are often implicated in healthcare-associated infections, which occur in settings such as hospitals and long-term care facilities. Surgical site infections, ventilator-associated pneumonia, and catheter-associated urinary tract infections are frequently caused by ESKAPEE pathogens [86].

Although the occurrence of ESKAPEE pathogens raises significant concerns in healthcare settings, these should not be disregarded when found in food and water sources as they can directly impact the hospital burden due to possible acquired infections and subsequent complications that may arise in humans. Furthermore, ESKAPEE pathogens are not necessarily considered foodborne, with the exceptions of some *E. coli* and *S. aureus* strains, but these can be established in the gut *via* food consumption, therefore serving as a source of resistance genes for the gut microbiota. Moreover, based on previously exemplified studies, a high prevalence of MDR in ESKAPEE pathogens in food and water sources has been reported. The interactions between animals and humans can subsequently transfer MDR ESKAPEE pathogens or resistance genes between each other or to other animals, humans or into the environments [79].

In this regard, the economic burden imposed by MDR ESKAPEE pathogens is substantial. The costs associated with treating infections caused by these pathogens are high due to the need for more expensive and potent antibiotics, prolonged hospital stays, and the additional medical care required for patients with severe infections. As a consequence of increased resistance acquired by these pathogens, the World Bank estimated that the impact of AMR would result in additional healthcare costs of US$1 trillion by 2050 and yearly Gross Domestic Product losses of US$1 trillion to US$3.4 trillion by 2030 [1,8,87].

## 6. Combating Antimicrobial Resistance in Food Production

Efforts to combat ESKAPEE pathogens in the food industry include surveillance, improved antibiotic stewardship, and the adoption of alternative measures to enhance animal health. The Food and Agriculture Organization (FAO) has developed the International FAO Antimicrobial Resistance Monitoring (InFARM) system [88], which aids countries in collecting, analysing, and utilising AMR data from livestock and aquaculture. This system integrates data into global surveillance networks, helping to monitor and manage AMR more effectively [87,88]. Additionally, the FAO’s Action Plan on Antimicrobial Resistance 2021–2025 focuses on strengthening governance, increasing awareness, and improving the use of antimicrobials in the food and agriculture sectors. This plan emphasises the importance of a One Health approach, which considers the interconnected health of humans, animals, and the environment to address the complex nature of AMR [88]. The graphical abstract indicates the ESKAPEE transmission through the One Health approach (created with BioRender [89]).

**Reduction of antibiotic use in food and feed-related sectors.** Increasing the ability of agriculture to meet societal needs for healthy, safe, and sustainable food, as well as reducing food waste and enhancing animal health and welfare, are some of the major issues the EU agriculture sector is currently facing [90]. The 2023–2027 Common Agricultural Policy (CAP) Strategic Plans of nearly all EU member states include measures for specific assistance to prevent antimicrobial resistance and to raise animal welfare standards during the ongoing programming term [91]. Integrating methods, investments, collaboration, and training will serve a broad variety of stakeholders [91]. Furthermore, to enhance the welfare of livestock animals and lessen the necessity of medicating them with antibiotics, EU farmers can apply for assistance in starting animal disease eradication programs or taking part in animal welfare labelling projects [90]. The legislative framework designed to safeguard water quality from potential pollutants associated with agricultural activities was established, with the management of manure from livestock and other fertilisers receiving specific consideration [92]. More funding for organic farming would contribute to lessening the need for antibiotics since organically raised animals must adhere to significantly stricter antibiotic usage guidelines than animals raised using traditional farming methods [90,91,93]. The Farm to Fork Strategy, a cornerstone of the European Union’s Green Deal, seeks to create a sustainable food system that ensures food security while promoting public health and environmental stewardship [93]. Launched in May 2020, the strategy outlines ambitious targets for reducing the environmental and climate footprint of the food system, including cutting the use of chemical pesticides by 50%, reducing nutrient losses by at least 50%, and decreasing the use of antimicrobials in agriculture and aquaculture by 50% by 2030 [93]. It also emphasises the importance of promoting sustainable farming practices, improving animal welfare, and fostering a circular economy to minimise food waste [93].

**Alternatives to reduce antibiotic dependence.** Alternative methods, such as probiotics or bacteriophages, have previously been addressed in livestock and food production. Probiotics are used to control infections by populating the gut microbiota of animals with beneficial organisms whereas, on the other hand, bacteriophages are used as biocontrol measures to reduce pathogenic microorganisms, therefore being viewed as a promising measure to reduce infections caused by the ESKAPEE pathogens [34,94,95].

**Food safety strategies.** Different strategies could be beneficial for the reduction of the burden of pathogenic microorganisms in food and food-related activities. For example, sanitation and cleaning protocols through regular and thorough cleaning and disinfection of equipment, surfaces, and facilities are essential to minimise microbial contamination [93]. This includes the use of effective sanitisers and adherence to cleaning schedules that ensure a hygienic processing environment [93]. Another important aspect to consider is personal hygiene and training, which ensure that food processing workers follow strict personal hygiene practices, such as proper handwashing, wearing protective clothing, and using appropriate equipment, activities meant to prevent cross-contamination [96]. Continuous training programs for employees on hygiene and AMR awareness are critical [96]. Lastly, implementing Hazard Analysis and Critical Control Points (HACCP) systems can help identify and control critical points where contamination with microorganisms might occur, ensuring that preventive measures are in place [97].

**Consumer education.** Educating consumers about the risks of AMR and the importance of responsible antibiotic use can influence purchasing decisions and promote the consumption of products obtained in antibiotic-free production systems. Furthermore, comprehensive consumer education initiatives can enhance public awareness, leading to informed choices that support both individual health and broader public health goals. Additionally, such educational efforts can create a culture of accountability among consumers, encouraging the adoption of more sustainable practices and reducing the overall demand for antibiotics in agriculture and healthcare [98].

## 7. Surveillance of ESKAPEE Pathogens in Clinical and Food Settings

Effective surveillance is the cornerstone of any strategy to combat AMR. Surveillance systems track the occurrence, patterns, and spread of AMR, providing crucial data for developing connected policies. It is also crucial for informing and tracking the effectiveness of regional, national, and international initiatives [8,99,100]. The WHO oversees the Global Antimicrobial Resistance and Use Surveillance System (GLASS) collaboration, which gathers, combines, and presents data from participating countries’ national surveillance systems on antimicrobial consumption and AMR. The goal of this system is to standardise the worldwide reporting of relevant and exceptional AMR and antimicrobial consumption data [100].

EARS-Net is a European-wide surveillance system coordinated by the European Centre for Disease Prevention and Control (ECDC) [99]. It collects data on AMR from participating countries, providing insights into the prevalence and trends of resistant pathogens [99]. As an example, EARS-Net Ireland, part of this network, monitors AMR in clinical isolates from invasive infections such as bloodstream and cerebrospinal fluid infections caused by key bacterial pathogens, including *E. coli*, *S. aureus*, *S. pneumoniae*, *E. faecium*, and *K. pneumoniae*, supporting the development of targeted interventions and policies to combat AMR in Ireland and across Europe. The surveillance system relies on a network of clinical microbiology laboratories across Ireland, which submit data on antimicrobial susceptibility testing results to a central database. This data is standardised to ensure consistency and reliability [96,99,101]. EARS-Net Ireland publishes annual reports that summarise AMR data, highlight key findings, and provide recommendations for healthcare providers and policymakers. These reports are essential for tracking progress and identifying areas needing improvement [99]. The data collected by EARS-Net Ireland is shared with ECDC and contributes to the broader European AMR surveillance efforts. This collaborative approach enhances the ability to monitor AMR trends at a continental level [99].

Regarding livestock and derived meat, there are national surveillance programmes that compile reports related to AMR and are further transmitted to the European Food Safety Authority (EFSA). These reports are jointly analysed by EFSA and ECDC and presented as an EU summary report every two years regarding AMR data on zoonotic and indicator bacteria from humans, animals, and food [102]. However, these reports focus on EU Member States, the UK, and some non-member states data and refer to particular AMR patterns in *Salmonella*, *E. coli*, ESBL-producing *E. coli* and *Salmonella*, *Campylobacter* and MRSA [102]. Moreover, non-governmental organisations such as the European Animal Health Study Center (CEESA), financed by the veterinary pharmaceutical sector, are monitoring indicator bacteria, AMR and drug usage in humans, livestock and derived meat through different surveillance programmes across Europe: VetPath (focuses on pathogens causing disease in animals), ComPath (AMR surveillance in companion animals), MycoPath (focuses on disease-causing mycoplasma in food-producing animals) and European Antimicrobial Susceptibility Surveillance in Animals (EASSA, which focuses on antimicrobial susceptibility of zoonotic and commensal bacteria in food animals) [103,104].

## 8. Conclusions and Looking Towards the Future

ESKAPEE pathogens are one of the most critical concerns from a clinical standpoint, primarily due to their extensive multidrug resistance profiles, including against last-resort antibiotics. Multiple factors contributing to this evolution pose an increasing challenge to preventing and controlling bacterial infections caused by the ESKAPEE group. The extensive usage of antibiotics in clinical practice and in animal husbandry, impacting foods and food-related activities, is anticipated to significantly affect future challenges, thereby putting substantial pressure on healthcare and healthcare-associated costs. In conclusion, changing attitudes towards antimicrobial stewardship is imperative, and combining efforts to fight antimicrobial resistance is much needed.

## Figures and Tables

**Table 1:** Multidrug resistance profiles of ESKAPEE pathogens highlight studies in which these profiles have been associated with certain foods.

Organism Target	Alternative Treatments	Resistance Determinants Encountered	Food Contamination	Reference
*E. faecium*	Linezolid, daptomycin, tigecyclines	**Beta-lactams:** PBP4/5 point mutations; altered cell wall; destruction of beta-lactam ring; production of beta-lactamases. **Glycopeptides:** Modified peptidoglycan cross-link target, which is encoded by *vanA*, *vanB*, *vanD*, *vanC*, *vanE*, and *vanG* genes. **Aminoglycosides:** low cell wall permeability; ribosome mutations, aminoglycoside-associated enzymes like Aph(2″), Ant(3″), Ant(6″). **Macrolides, Lincosamides, Streptogramins, Pleuromutilins (MLSPs):** ABC efflux pumps-streptogramin resistance: *msr*C gene; altered ribosomes-*erm*B gene which modifies 23S rRNA; rRNA point mutations contributing to linezolid resistance. **Tetracyclines:** efflux pumps due to *tet*M and *tet*L genes. **Phenicols:** *cat* genes leading to inactivation of chloramphenicol.	Beef, poultry, pork, cheese, fermented milk	[28,35,36,37,55,56,57]
*S. aureus*	Vancomycin, linezolid, daptomycin	**Beta-lactams:***mec*A and *mec*C genes through an altered PBP2a target; production of beta-lactamases: *blaZ* gene involved. **Aminoglycosides:** *aac*, *aph* and *ant* genes through acetylating and/or phosphorylating enzymes (e.g. Ant(4′)-IA, Aph(3′)-III). **Tetracyclines:** efflux pumps; *tet*K, *tet*M, *tet*L genes; ribosomal safeguarding; chromosomal or transposon-located *tet*M or *tet*O elements. **Glycopeptides:** drug inactivation, *van*A gene role through modified targets. **Phenicols:** *cat* genes leading to inactivation of chloramphenicol. **MLSPs:** different enzymes involved in the modification of the drug.	Cheese, dairy products, raw meat, frozen meat, RTE meat	[38,39,57,58,59,60]
*K. pneumoniae*	Polymyxins, ceftazidime-avibactam, tigecyclines	**Beta-lactams:** enzymatic drug inactivation or modification due to the production of ESBLs and carbapenemases, alteration of PBPs (*pbp2* and *pbp4*). **Aminoglycosides:** increased efflux pump expression; involvement of aminoglycoside-modifying enzymes (AMEs). F**luoroquinolones:** *qnr*A, *qnr*B, and *qnr*S genes through plasmid-mediated mechanisms; efflux pumps expressing genes, including *qep*A and *oqx*AB. **Polymyxins:** *mgr*B, *phoPQ*, *pmr*A, and *pmr*D genes. **Phenicols:** *cat* genes leading to inactivation of chloramphenicol. **Tigecyclines:** efflux pump systems such as AcrAB-TolC and *Oqx*AB, 16S rRNA (e.g., *rrs* gene) or ribosomal proteins (e.g., *rpsJ* gene).	Turkey, fish, cattle and chicken meats, milk, raw fresh vegetables	[41,42,43,57,61,62,63,64]
*A. baumannii*	Polymyxins, tigecyclines, carbapenems	**Beta-lactams:** inactivation of antibiotic target; increase of efflux pumps (*ade* gene cluster), production of different beta-lactamases such as IMP, VIM, NDM, SIM; resistance genes such as *bla*_OXA_-23, *bla*_OXA_-51, *bla*_OXA_-58, *bla*_TEM_ and *bla*_CTX_-M; alterations in outer membrane proteins. **Aminoglycosides:** AAC(6′)-Ib and ANT(2″)-Ia enzymes; efflux pumps; AdeABC and AdeIJK. **Sulfonamides:** *sul*1 and *sul*2; efflux pumps, *Mex*AB-*Opr*M. **Tetracyclines:** efflux pumps, AdeABC; t*et*A and t*et*B genes. **Phenicols:** inactivation of chloramphenicol by the action of chloramphenicol acyltransferase enzymes. **Polymyxins:** *mcr-*1 gene harbouring colistin resistance, LPS lipid A modification; mutations of the *lpx*A, *lpx*C, and *lpx*D genes, *lps*B, *lpt*D, and *vac*J expression.	Fruits and vegetables, raw milk, meat products	[19,44,45,57,65,66]
*P. aeruginosa*	Polymyxins, ceftolozane-tazobactam, cefiderocol	**Beta-lactams:** chromosomal AmpC synthesis with porin modification; efflux pumps encoded by *mex*A-*mex*B-*opr*M and *mex*XY genes. **Carbapenems:** deficiency of the OprD protein leading to reduced permeability; carbapenem hydrolysing non-metallo-beta-lactamases such as KPC, SME, GES, IMI-1. **Aminoglycosides:** AMEs (*aac(6′)-Ib*, *aphA1*, and *aadB* genes). **Phenicols:** *cat* genes leading to inactivation of chloramphenicol. **Tetracyclines:** efflux pumps (*tetR*, *lysR*, *marR*, and *araC* genes).	Milk and dairy products, fruits and vegetables, cold chain meat products	[28,47,48,57,67,68]
*Enterobacter* spp.	Ceftazidime-avibactam, polymyxins, tigecyclines	**Beta-lactams:** production of different enzymes such as VIM, OXA, MBL-1, and KPC, AmpC; alteration of PBPs (*pbp3* gene). **Aminoglycosides:** ribosomal modification due to *rmtE* gene. **Phenicols:** efflux pumps *AcrAB–TolC* and *eefABC.* **Tetracyclines:** *AcrAB–TolC* and *eefABC* efflux pumps.	Yoghurt, cheese, beef, chicken, milk	[28,50,51]
*E. coli*	Ceftazidime-avibactam, polymyxins, tigecyclines	**Beta-lactams:***bla*_CTX-M_, ESBLs ability to hydrolyse cephalosporins, monobactams and classical penicillins. **Polymyxins:***mcr-*1 gene harbouring colistin resistance. **Fosfomycins:***fos*A3 resistance gene	Animal origin foods, lettuce	[20,22,52,54]

## Data Availability

The data presented in the study are available in public accessible repositories.
